# Supplementary material for: Dynamic restructuring of nickel sulfides for electrocatalytic hydrogen evolution reaction
Source: Nat Commun. 2024 Jun 24;15:5336. doi: 10.1038/s41467-024-49015-4 (PMC11196257; doi:10.1038/s41467-024-49015-4)
Supplement: Supplementary file 1 — Supplementary Information [file 41467_2024_49015_MOESM1_ESM.pdf]

Supplementary Information for
Dynamic restructuring of nickel sulfides for electrocatalytic hydrogen evolution reaction

Xingyu Ding[1,2]†, Da Liu[2]†, Pengju Zhao[1], Xing Chen[1], Hongxia Wang[1], Freddy E. Oropeza[3*], Giulio Gorni[4,5], Mariam Barawi[3], Miguel García-Tecedor[3], Víctor A. de la Peña O'Shea[3], Jan P. Hofmann[6], Jianfeng Li[1], Jongkyoung Kim[7], Seungho Cho[7], Renbing Wu[2*], Kelvin H. L. Zhang[1*]

[1]*State Key Laboratory of Physical Chemistry of Solid Surfaces, College of Chemistry and Chemical Engineering, Xiamen University, Xiamen 361005, China*

[2]*Department of Materials Science, Fudan University, Shanghai 200433, China*

[3]*Photoactivated Processes Unit, IMDEA Energy Institute, Parque Tecnológico de Móstoles, Avda. Ramón de la Sagra 3, 28935 Móstoles, Madrid, Spain*

[4]*Laser Processing Group, Institute of Optics (CSIC), C/Serrano 121, 28006 Madrid, Spain*

[5]*CELLS-ALBA Synchrotron, Carrer de la Llum 2-26, 08290 Cerdanyola del Vallès, Spain*

[6]*Surface Science Laboratory, Department of Materials and Earth Sciences, Technical University of Darmstadt, Otto-Berndt-Strasse 3, 64287 Darmstadt, Germany*

[7]*Department of Materials Science and Engineering, Ulsan National Institute of Science and Technology (UNIST), Ulsan, 44919, Republic of Korea*

†*Xingyu Ding and Da Liu contributed equally to this work.*

*Email: freddy.oropeza@imdea.org; rbwu@fudan.edu.cn; kelvinzhang@xmu.edu.cn

Supplementary Figures and Tables

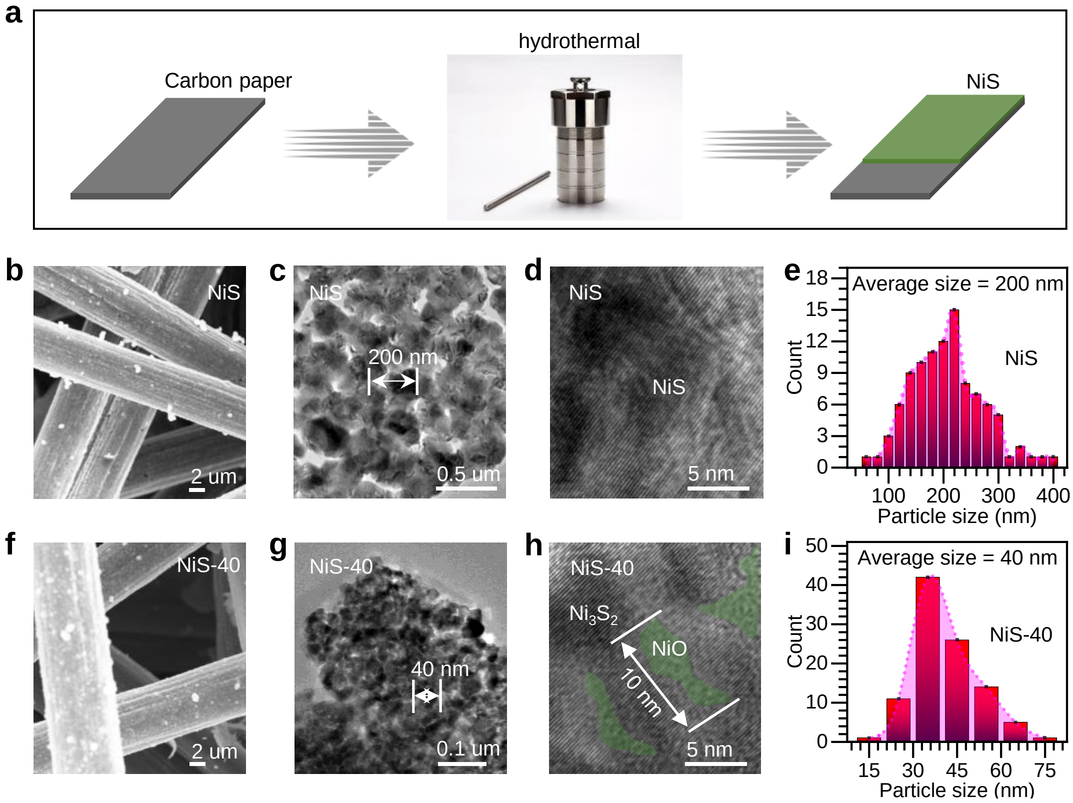

Supplementary Fig.1 | Material preparation and electron microscopy characterization. **a** Schematic illustration of the synthesis process of NiS on carbon paper. **b** SEM image of the as-synthesized NiS on carbon paper. **c** TEM image of the as-synthesized NiS, and **d** the corresponding high-resolution TEM image. **e** The statistics of NiS particle size distribution. **f** SEM image of the NiS-40 (Ni_3S_2/NiO) sample. **g** TEM image of the NiS-40, and **h** the corresponding high-resolution TEM image. **i** The statistics of particle size distribution of the NiS-40 sample.

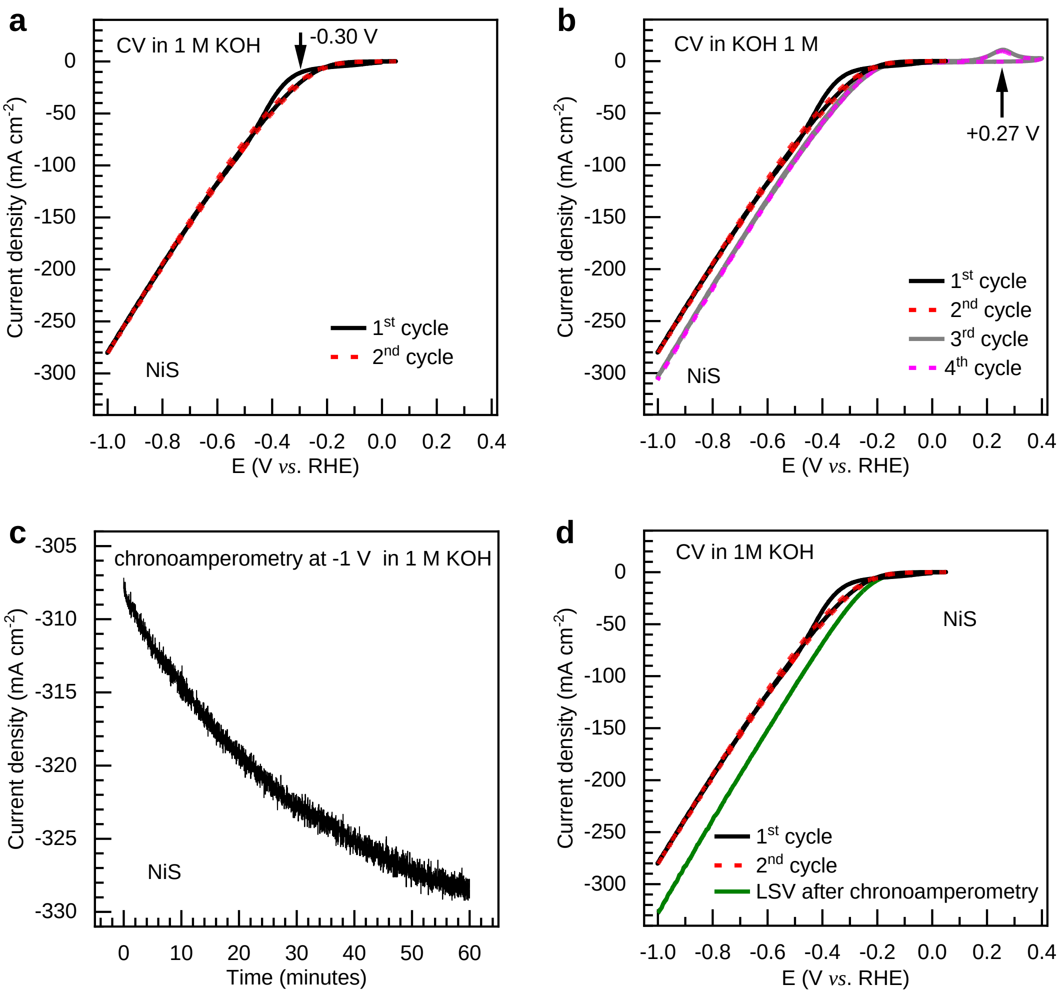

Supplementary Fig. 2 | Electrochemical measurement based on different activation strategies. a Cyclic voltammetry (CV) measurements for the 1st and 2nd cycles with an upper limit of the potential at 0.05 V *vs*. RHE. **b** CV measurements for the 1st, 2nd cycles with an upper limit of the potential at 0.05 V *vs*. RHE and the 3rd and 4th cycles with an upper limit of the potential at 0.40 V *vs*. RHE. **c** Chronoamperometry of NiS sample measured under a constant potential of −1.00 V *vs*. RHE. **d** The comparison between linear sweep voltammetry (LSV) of NiS after the chronoamperometry measurement and the 1st, 2nd CV cycles.

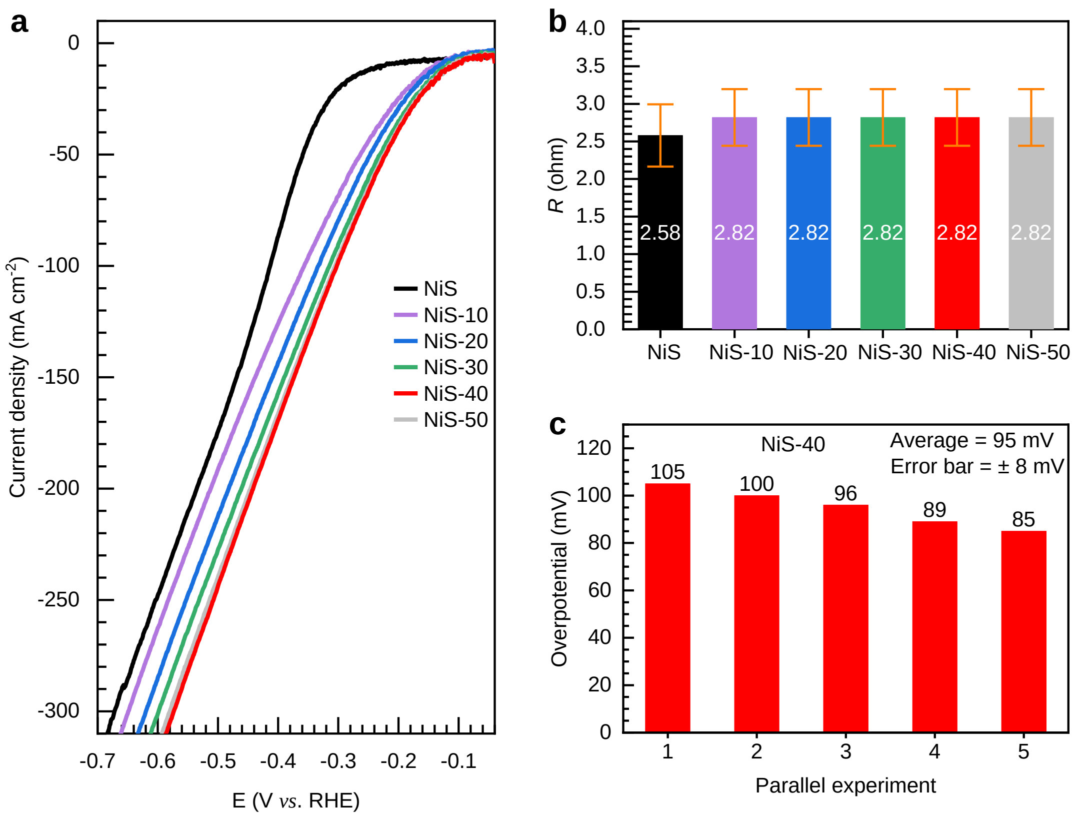

Supplementary Fig. 3 | Electrochemical measurement, HER activity evaluation and the value of contact resistance (*R*). a Non-*iR* corrected Linear sweep voltammetry (LSV) polarization curves of as-synthesized NiS, NiS-10, NiS-20, NiS-30, NiS-40 and NiS-50 samples measured in 1 M KOH solution. NiS-10, NiS-20, NiS-30, NiS-40 and NiS-50 are NiS samples after 10-minute, 20-minute, 30-minute, 40-minute and 50-minute chronoamperometry measurements at −1.00 V *vs*. RHE, respectively. **b** The contact resistance (*R*) during assembly of those NiS, NiS-10, NiS-20, NiS-30, NiS-40 and NiS-50 samples in the electrochemical cell. **c** The value of overpotential at 10 mA cm^{-2} in 5 parallel experiments of NiS-40 sample.

[**Note:** In Supplementary Fig. 3b, each sample underwent 4 tests for contact resistance. The obtained average *R* was used to correct E in Supplementary Fig. 3a. The E_{cor} in Fig. 1c can be obtained by using 85% *iR* to correct E in Supplementary Fig. 3a. As shown in Supplementary Fig. 3c, 5 independent LSV measurements were conducted to NiS-40. After statistical calculation, the average overpotential was 95 mV with an error bar of ±8 mV.]

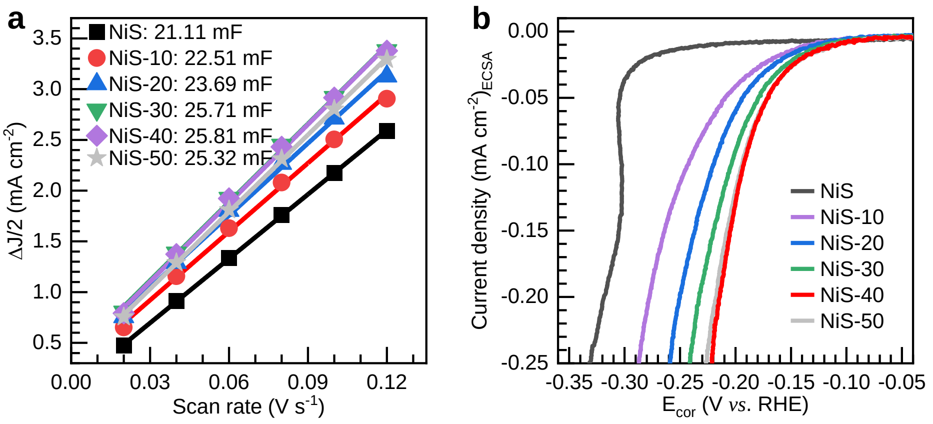

Supplementary Fig. 4 | Assessment of HER activity normalized by ECSA. a Plot of current density v.s. scan rates extracted from Supplementary Fig. 5. The slope is double layer capacitance (C_{dl}) and the electrochemical active surface area (ECSA) can be calculated from C_{dl} according to the equation: ECSA = C_{dl}/C_s. Where C_s is usually equal to 0.04 mF cm^{-2} in 1 M KOH electrolyte[1]. **b** Linear sweep voltammetry (LSV) polarization curves normalized by ECSA of as-synthesized NiS, NiS-10, NiS-20, NiS-30, NiS-40 and NiS-50 samples.

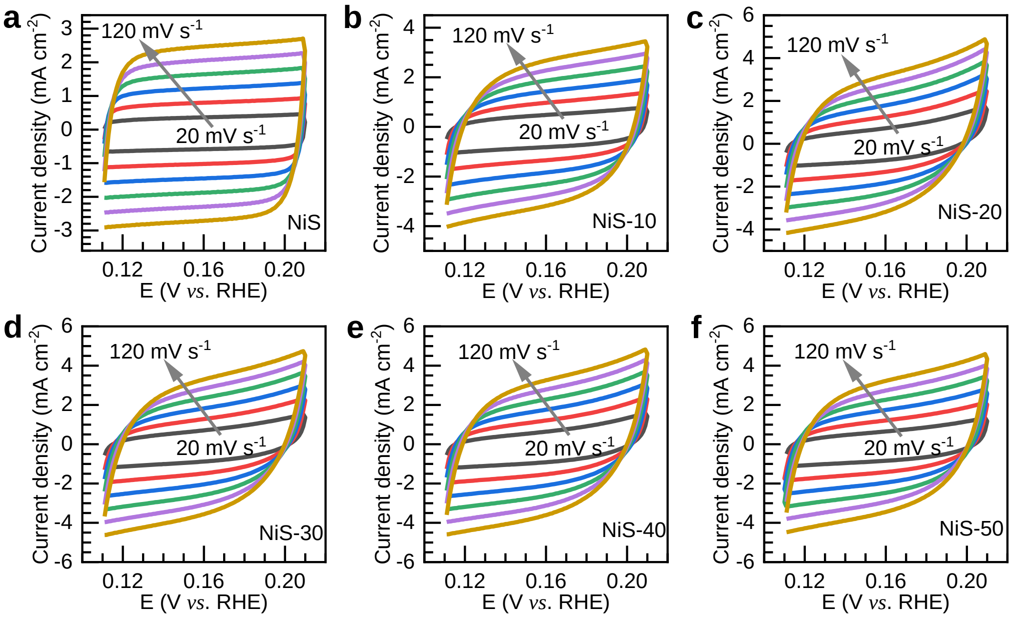

Supplementary Fig. 5 | Electrochemical measurement for the determination of ECSA. Cyclic voltammogram of curves of **a** NiS, **b** NiS-10, **c** NiS-20, **d** NiS-30, **e** NiS-40 and **f** NiS-50 with different scan rates (0.02, 0.04, 0.06, 0.08, 0.10 and 0.12 V s^{-1}).

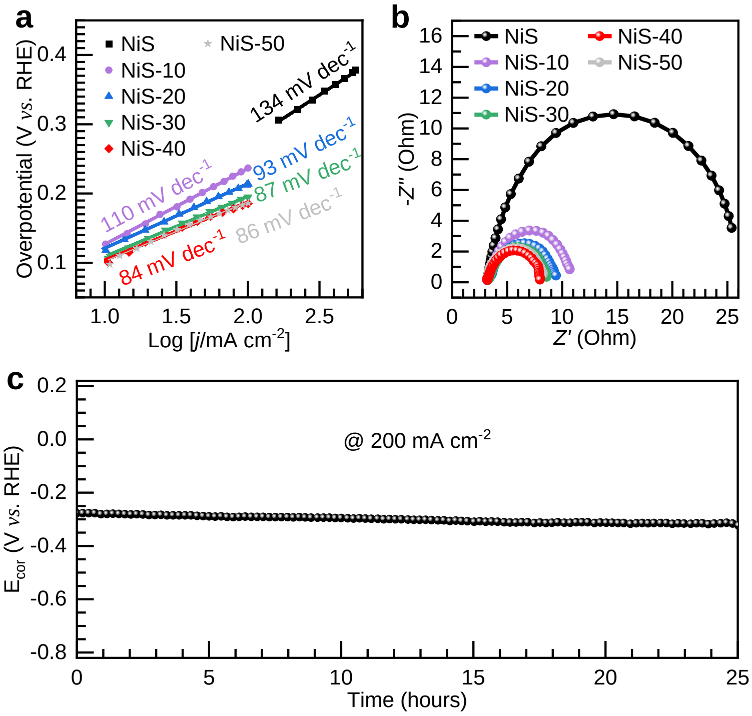

Supplementary Fig. 6 | Electrochemical measurements for Tafel slope, EIS and stability. a The Tafel curves of NiS, NiS-10, NiS-20, NiS-30, NiS-40 and NiS-50. **b** Nyquist plots of EIS measurements at −0.20 V *vs*. RHE in a sweep frequency range of 100 kHz to 0.1 Hz. **c** Chronopotentiometric curves of NiS measured under a constant current density of 200 mA cm^{-2}.

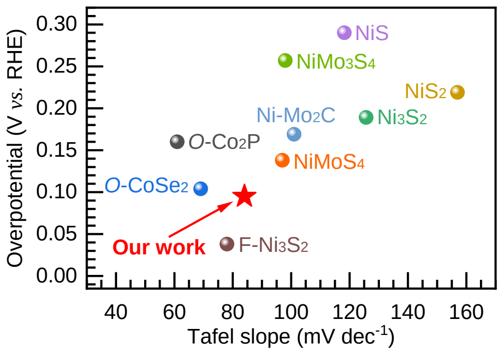

Supplementary Fig. 7 | Comparison of our catalytic performance to that in the literature. Comparison of Tafel slope and overpotential for various non-noble-metal-based HER catalysts in alkaline electrolytes (Supplementary Table 1).

Supplementary Table 1 | Comparison of catalytic parameters of our catalyst and other non-noble-metal-based HER catalysts in alkaline electrolytes.

Catalyst	Electrolyte	Overpotential @ 10 mA cm^{-2} (mV *vs.* RHE)	Tafel slope (mV dec^{-1})	Reference
O-Co_2P	1 M KOH	160	61	[2]
$NiMo_3S_4$	0.1 M KOH	257	98	[3]
O-$CoSe_2$	1 M KOH	104	69	[4]
Ni_3S_2	1 M KOH	189	125.7	[5]
NiS	1 M KOH	290	118.2	[6]
NiS_2	1 M KOH	219	157	[7]
Co-NiS_2	1 M KOH	80	43	[8]
F-Ni_3S_2	1 M KOH	38	78	[9]
CoS_2	0.5 M H_2SO_4	145	51.6	[10]
$NiMoS_4$	1 M KOH	138	97	[11]
Ni-Mo_2C	1 M KOH	169	101	[12]
Our work	1 M KOH	95	84	-

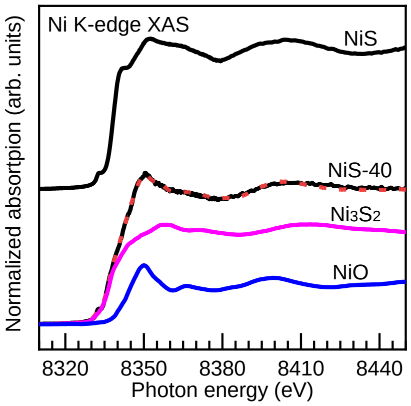

Supplementary Fig. 8 | Identification of phase composition before and after the HER via XAS analysis. The Ni K-edge XAS of NiS and NiS-40 along with Ni_3S_2 and NiO as reference. The Ni K-edge XAS of NiS-40 consists of a linear combination of spectra of Ni_3S_2 and NiO.

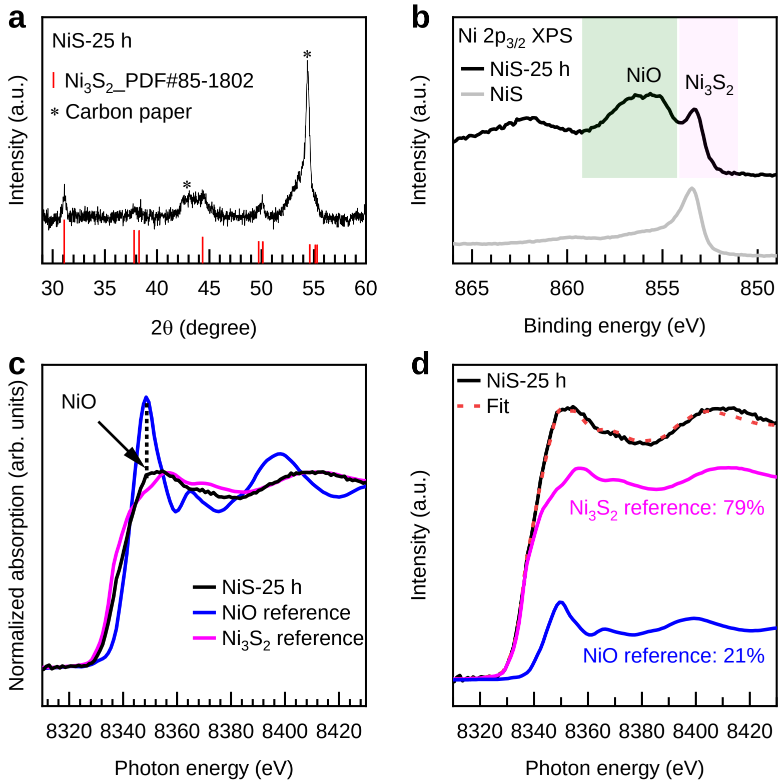

Supplementary Fig. 9 | The characterization of the HER-post catalyst after conducting 25-hour chronopotentiometric measurement at a current density of 200 mA cm^{-2}. **a** XRD pattern; **b** Ni 2$p_{3/2}$ XPS spectra; **c** Ni K-edge XAS spectra; **d** the Ni K-edge XAS of HER-post catalyst consists of a linear combination of spectra of Ni_3S_2 and NiO reference. [**Note:** As shown in Supplementary Figs. 9a and 9b, the XRD patterns of HER-post catalyst confirm that the pristine NiS transforms into Ni_3S_2, while the Ni 2$p_{3/2}$ XPS spectra show obvious NiO features. The Ni K-edge XAS spectra further show the NiO characteristic feature located at 8350 eV (Supplementary Fig. 9c), and it can be fitted by using NiO and Ni_3S_2 reference well (Supplementary Fig. 9d). The above characterizations strongly evidence that the HER-post catalysts exist in form of Ni_3S_2/NiO.]

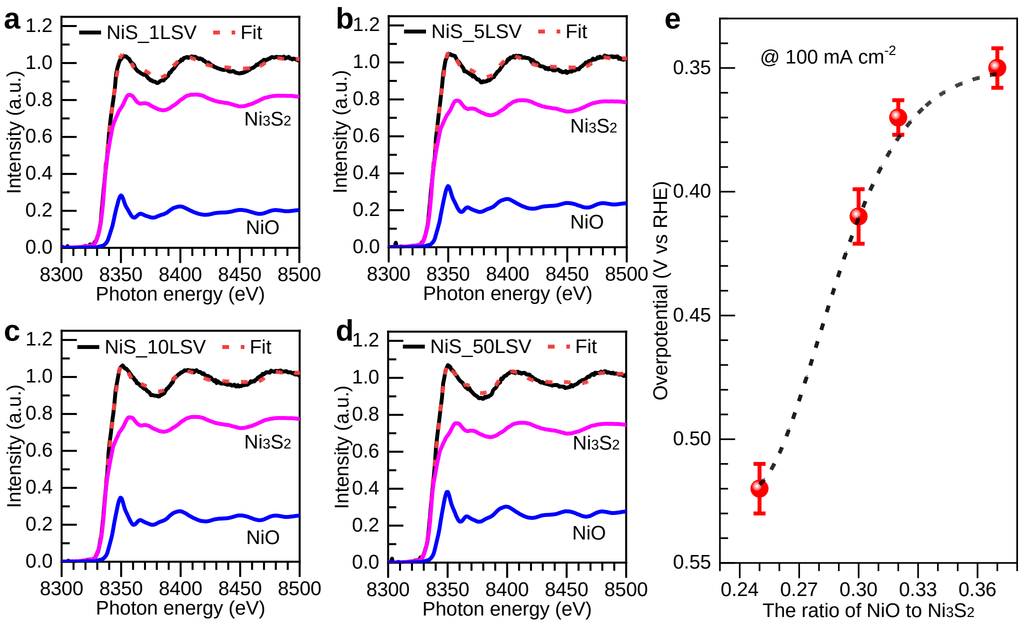

Supplementary Fig. 10 | Quantitative component analysis based on XAS analysis. The Ni K-edge XAS spectra and their linear combination for NiS catalyst after **a** 1 LSV, **b** 5 LSV, **c** 10 LSV and **d** 50 LSV measurements. The components obtained by linear combination are shown in Supplementary Table 2. **e** The overpotential as a function of NiO to Ni_3S_2 ratio. The results show that HER activity is positively correlated with the amount of NiO.

Supplementary Table 2 | The result of composition for NiS after conducting 1, 5, 10 and 50 LSV measurements.

Sample	NiO (%)	Ni_3S_2 (%)	R-factor	The ratio of NiO to Ni_3S_2
**NiS_1LSV**	20.2±0.9	79.3±1.0	0.0009	0.25
**NiS_5LSV**	24.0±0.9	76.7±1.0	0.0008	0.31
**NiS_10LSV**	24.1±1.0	75.6±1.0	0.0010	0.32
**NiS_50LSV**	27.0±0.9	73.1±1.0	0.0007	0.37

The compositions are obtained by using Ni_3S_2 and NiO as references to conduct a linear combination on the Ni K-edge XAS spectra as shown in Supplementary Figs. 10a–d.

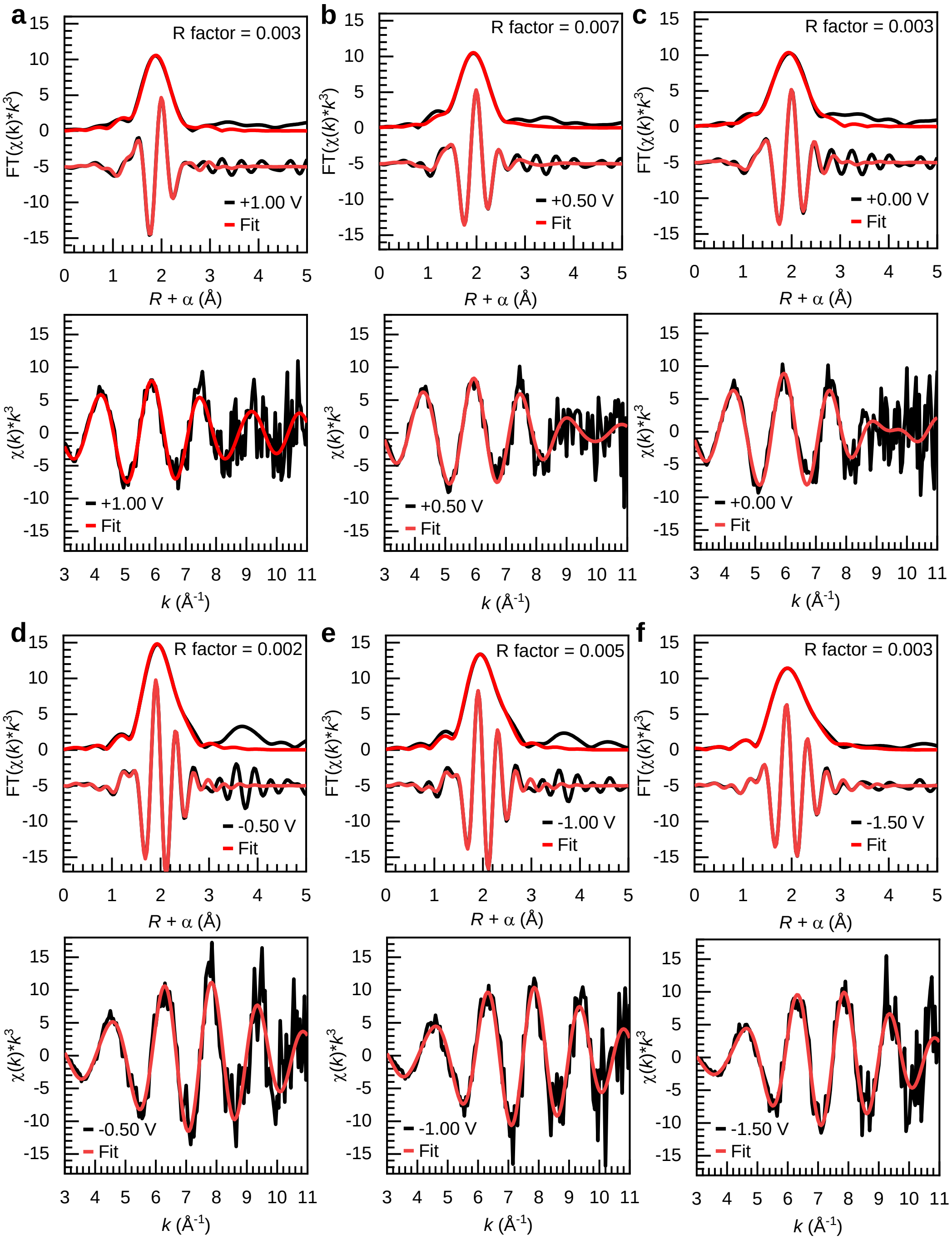

Supplementary Fig. 11 | Operando XAS analysis of fine structures. The operando FT-EXAFS, the imaginary part of FT-EXAFS, EXAFS in *k*-space and their fitting of NiS at different applied voltages (**a** +1.00, **b** +0.50, **c** 0.00, **d** −0.50, **e** −1.00 and **f** −1.50 V *vs.* RHE) during the stage of the negative sweep of CV. The fitting results are shown in Supplementary Table 3.

Supplementary Table 3 | Operando EXAFS fitting parameters at the Ni K-edge of NiS at different applied voltages during the stage of negative sweep of CV. (S_0^2 = 0.8)

**Applied voltage (V *vs*. RHE)**	**Path**	**CN**	***R* (Å)**	**σ^2 (Å^2)**	**R-factor**
**+1.00 (region I)**	Ni-S	5.3±0.8	2.344±0.018	0.005±0.003	0.003
	Ni-Ni	2.00*	2.70±0.03	0.009±0.004	
**+0.50 (region I)**	Ni-S	6±1	2.352±0.017	0.009±0.003	0.007
	Ni-Ni	2.00*	2.68±0.03	0.009±0.004	
**0.00 (region I)**	Ni-S	6±1	2.35±0.02	0.008±0.003	0.003
	Ni-Ni	2.00*	2.68±0.02	0.005±0.003	
**−0.50 (region II)**	Ni-S	4.3±0.8	2.271±0.017	0.007±0.002	0.002
	Ni-Ni	4.00*	2.51±0.02	0.007±0.004	
**−1.00 (region II)**	Ni-S	4.3±0.9	2.28±0.02	0.005±0.001	0.005
	Ni-Ni	4.00*	2.505±0.015	0.010±0.002	
**−1.50 (region II)**	Ni-S	3.3±0.7	2.26±0.02	0.005±0.002	0.003
	Ni-Ni	4.00*	2.507±0.013	0.007±0.002	

CN: coordination number. ***R*:** bond distance. **σ^2:** Debye-Waller factors. **R factor:** goodness of fit. The asterisks (*) mean that the parameter has been kept fixed during the fitting process.

[**Note:** For the negative sweep of CV, the results indicate the phase change from NiS to Ni_3S_2. At positive potentials, the Ni-S coordination is consistent with 6 and the bond distance of Ni-S is around 2.35 Å. The contribution from Ni-Ni scattering is also observed around 2.68 Å. These values are in good agreement with the structure of NiS. At negative potentials, the Ni-S coordination number is consistent with 4 and the bond length is around 2.27 Å in agreement with the structure of Ni_3S_2. The Ni-Ni shell is also 4-coordinated and around 2.51 Å, similar to Ni_3S_2.]

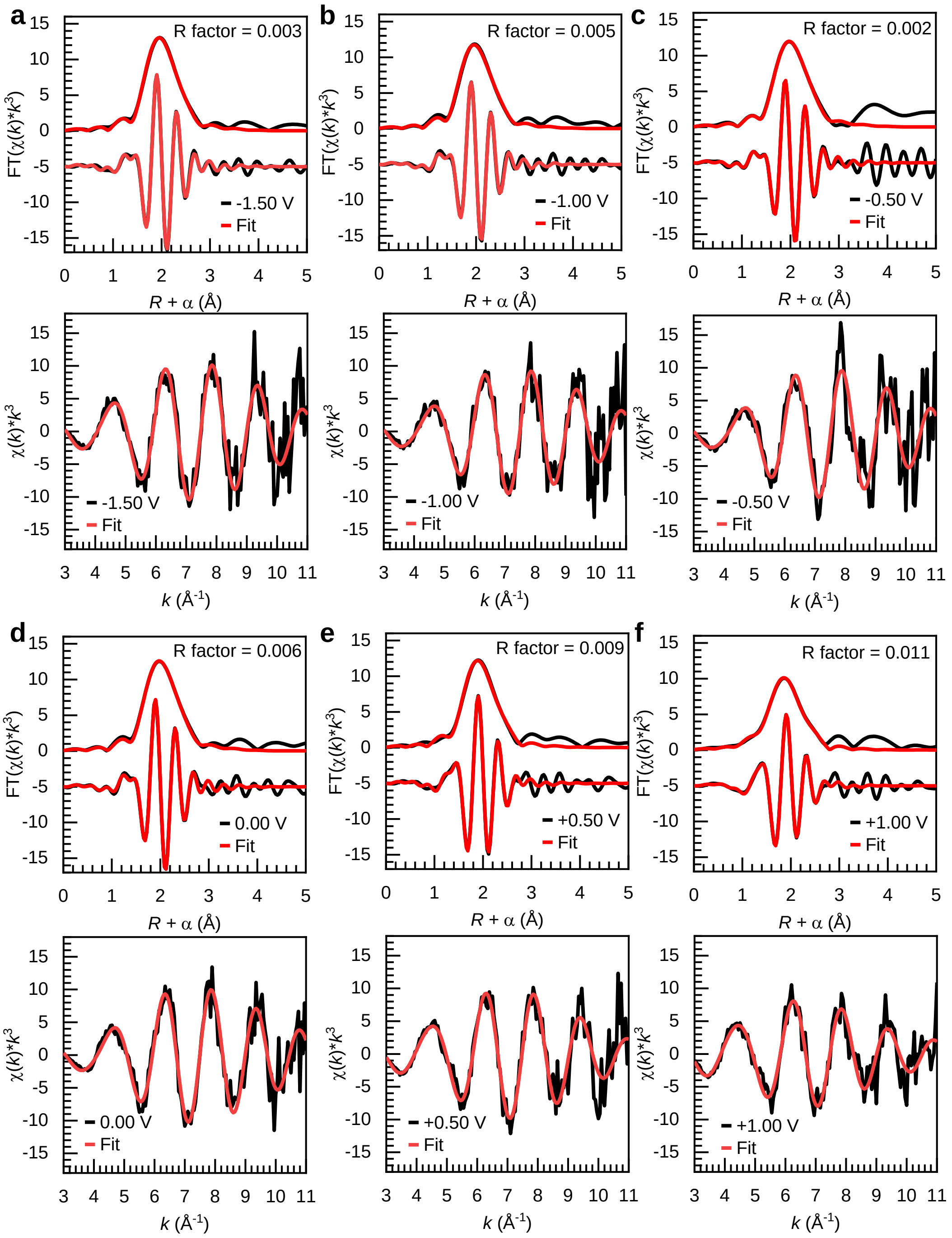

Supplementary Fig. 12 | Operando XAS analysis of fine structures. The operando FT-EXAFS, the imaginary part of FT-EXAFS, EXAFS in *k*-space and their fitting of NiS at different applied voltages (**a** −1.50, **b** −1.00, **c** −0.50, **d** 0.00, **e** +0.50 and **f** +1.00 V *vs.* RHE) during the stage of positive sweep of CV. The fitting results are shown in Supplementary Table 4.

Supplementary Table 4 | Operando EXAFS fitting parameters at the Ni K-edge of NiS in different applied voltages during the stage of positive sweep of CV. (S_0^2 = 0.8)

Applied voltage (V *vs*. RHE)	Path	CN	*R* (Å)	σ^2 ($Å^2$)	R-factor
**−1.50 (region II)**	Ni-S	3.3±0.6	2.262±0.013	0.005±0.003	0.003
	Ni-Ni	4.00*	2.50±0.01	0.007±0.001	
**−1.00 (region II)**	Ni-S	2.9±0.8	2.262±0.016	0.005±0.004	0.005
	Ni-Ni	4.00*	2.502±0.012	0.007±0.001	
**−0.50 (region II)**	Ni-S	2.9±0.6	2.258±0.012	0.006±0.001	0.002
	Ni-Ni	4.00*	2.495±0.008	0.007±0.001	
**0.00 (region II)**	Ni-S	3.0±0.6	2.258±0.017	0.006±0.003	0.006
	Ni-Ni	4.00*	2.50±0.01	0.006±0.001	
**+0.50 (region III)**	Ni-S	4 (x=0.87)	2.267±0.015	0.005±0.001	0.009
	Ni-Ni	4.00*	2.509±0.015	0.010±0.002	
	Ni-O	6 (1−x=0.13)	2.03±0.05	0.005±0.001	
**+1.00 (region III)**	Ni-S	4 (x=0.78)	2.286±0.014	0.005±0.002	0.011
	Ni-Ni	4.00*	2.51±0.002	0.013±0.002	
	Ni-O	6(1−x=0.22)	2.00±0.065	0.005±0.002	

CN: coordination number. ***R*:** bond distance. **σ^2:** Debye-Waller factors. **R factor:** goodness of fit. The asterisks (*) mean that the parameter has been kept fixed during the fitting process. The 'x' and '1−x' represent the weight of Ni-S path and Ni-O path, respectively.

[**Note:** For the positive sweep of CV, the results indicate that at more negative potentials the local structure is similar to that of Ni_3S_2 with a slightly lower coordination number of Ni-S compared to the crystallographic data, which, as discussed in the manuscript, might indicate the presence of S vacancies. The Ni-S bond distance is around 2.26 Å, which agrees with that of Ni_3S_2. At positive potentials, there is a clear shrink of the bond length associated to the partial formation of Ni-O bonds at around 2.00 Å. The weight of the Ni-O bond compared to Ni-S is around 13% and 22% at +0.50 and +1.00 V *vs*. RHE respectively (see region III in Supplementary Table 4), in agreement with the values (13%

and 22%) obtained from the linear combination fitting of XANES spectra, as shown in Fig. 2c and Supplementary Table 5.]

Supplementary Table 5 | The result of composition for NiS during operando XAS at +0.50 and +1.00 V *vs*. RHE. This corresponds to the spectra in region III of Fig. 2c. The uncertainty on the NiO and Ni_3S_2 was estimated considering the spectral deviations of 4–5 repetitions and the deviation is around 1–2%.

Applied potential (V *vs*. RHE)	NiO (%)	Ni_3S_2 (%)	R-factor
**+0.50**	13±0.3	87±1.7	0.0006716
**+1.00**	22±0.5	78±1.6	0.0004868

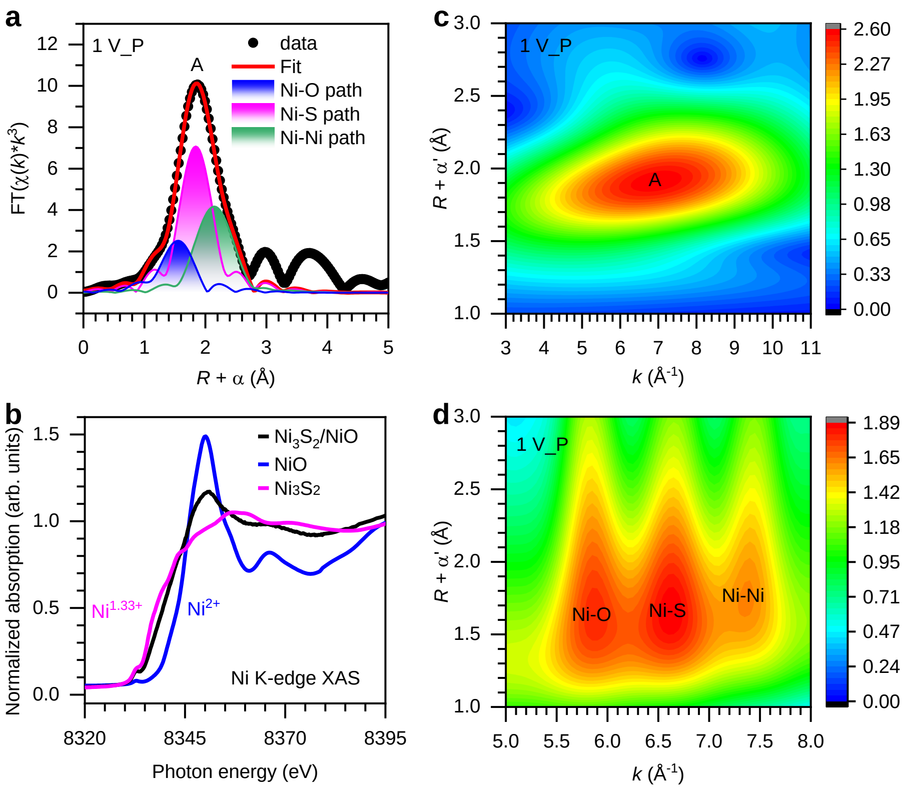

Supplementary Fig. 13 | The analysis of Wavelet transform. a The FT-EXAFS obtained by the Fourier transformation of Ni K-edge EXAFS spectra at +1.00 V in region III in Fig. 2c, and its fitting by using Ni-O, Ni-S and Ni-Ni paths. **b** The Ni K-edge XAS spectra of Ni_3S_2/NiO at +1.00 V, along with NiO and Ni_3S_2 as references. The results

indicate the oxidation state of Ni in Ni_3S_2/NiO is between +1.33 and +2. **c** Overview Wavelet transform (WT) of the Ni K-edge EXAFS spectra with η=10, σ=1, 3 $Å^{-1} \leqslant k \leqslant$ 11 $Å^{-1}$. **d** High-resolution WT of the first shell with η=1.6, σ=1, 5 $Å^{-1} \leqslant k \leqslant$ 11 $Å^{-1}$.

[**Note:** According to PCA analysis (Supplementary Note 4), we use Ni-O, Ni-S and Ni-Ni scattering paths to fit the data, and the results of fitting were reliable because of the reasonable parameter as shown in region III in Supplementary Table 4. Because these O, S and Ni backscattering are difficult to distinguish in the first shell as shown in Supplementary Fig. 13a, we use wavelet transform to resolve them along *k*. Supplementary Fig. 13c shows the overview wavelet in the interval 3 $Å^{-1} \leqslant k \leqslant$ 11 $Å^{-1}$ with Morlet parameters η=10, σ=1, and a signal weighting of k^3. Only one main peak A in Supplementary Fig. 13c agrees well with the FT-EXAFS in Supplementary Fig. 13a. By optimizing parameters η=1.6, σ=1 in the interval 5 $Å^{-1} \leqslant k \leqslant$ 8 $Å^{-1}$, the resolution along *k* is improved and the one main peak is resolved to three peaks at *k*=5.9, 6.6 and 7.4 $Å^{-1}$, respectively, thereby revealing the presence of Ni-O, Ni-S and Ni-Ni in the first shell (see Supplementary Fig. 13d).]

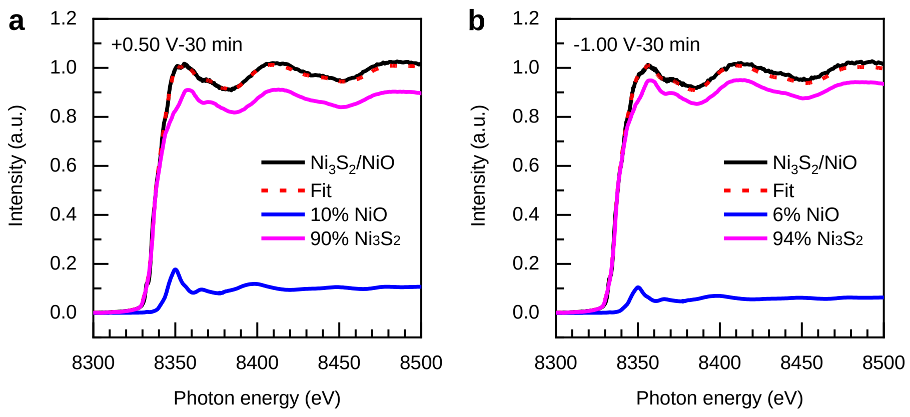

Supplementary Fig. 14 | Quantitative component analysis based on XAS analysis. Operando XAS at Ni K-edge of a NiS electrode in chronoamperometry for 30 minutes at an applied potential of **a** +0.50 V and **b** −1.00 V *vs.* RHE, and their linear combination along with NiO and Ni_3S_2 as reference. The components obtained by linear combination are shown in Supplementary Table 6.

Supplementary Table 6 | The results of composition for NiS after conducting chronoamperometry for 30 minutes at an applied potential of +0.50 V and −1.00 V *vs.* RHE.

Applied potential (V *vs.* RHE)	NiO (%)	Ni_3S_2 (%)	R-factor
**+0.50**	10±0.2	90±1.8	0.0004609
**−1.00**	6±0.1	94±1.9	0.0004288

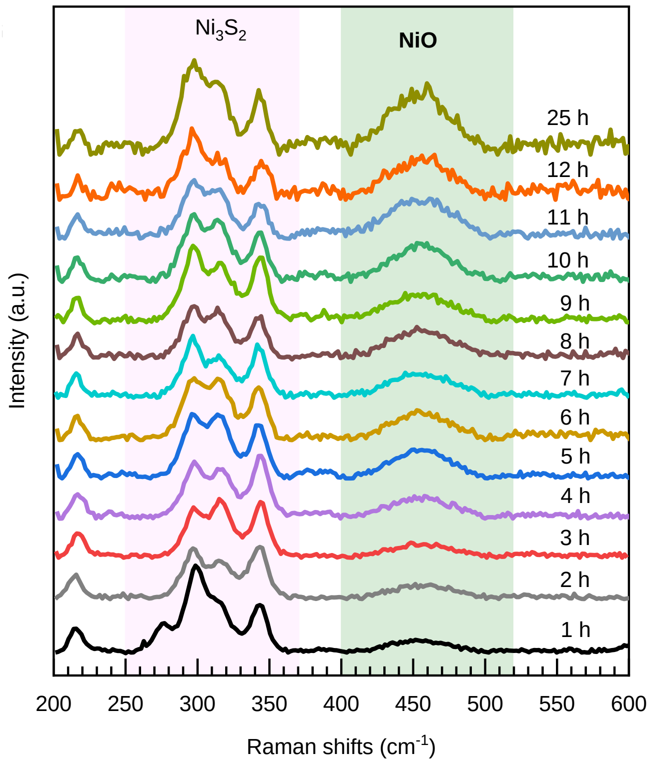

Supplementary Fig. 15 | Operando Raman measurement during long-term HER measurement. Operando Raman spectra of NiS electrode in CA measurement for 25 hours at a constant applied potential of −0.3 V *vs.* RHE.

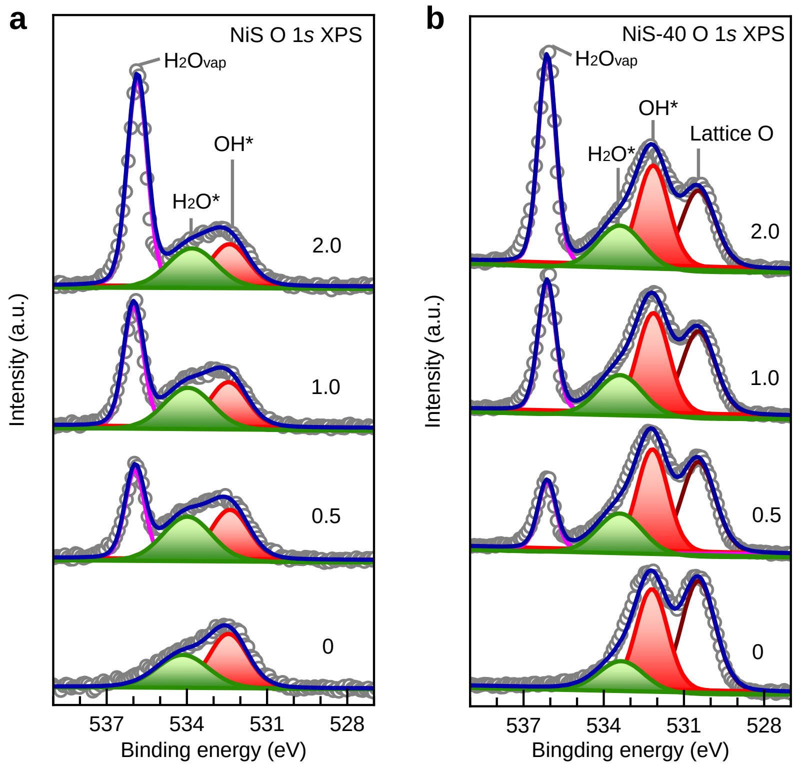

Supplementary Fig. 16 | NAP-XPS measurement under different water vapor pressures. O 1*s* NAP-XPS spectra and peak fittings for **a** NiS (marked as NiS), and **b** NiS after HER measurement (marked as NiS-40, *i.e.*, Ni_3S_2/NiO). The XPS measurements are conducted in different water pressures of 0, 0.5, 1.0, and 2.0 mbar. The fitting results are shown in Supplementary Table 7.

Supplementary Table 7 | Quantification of the lattice O, OH*, H_2O* and H_2O_{vap} through the fitting of NAP-XPS spectra (Supplementary Fig. 16), and the value of OH*/(OH* + H_2O*) is calculated.

**Sample**	**Water pressure**	**Area from fitting NAP-XPS**				**R_{dis} = OH*/(OH*+H_2O*)**
		**Lattice O**	**OH***	**H_2O***	**H_2O_{vap}**	
**NiS**	0.0	0.00	1.70	1.42	0.00	0.545
	0.5	0.00	1.53	1.81	1.43	0.458
	1.0	0.00	1.52	1.85	2.15	0.451
	2.0	0.00	1.41	1.76	3.70	0.445
**NiS-40**	0.0	1.67	1.44	0.64	0.00	0.692
	0.5	1.38	1.43	0.85	0.54	0.627
	1.0	1.27	1.44	0.86	1.03	0.626
	2.0	1.18	1.45	0.92	1.65	0.612

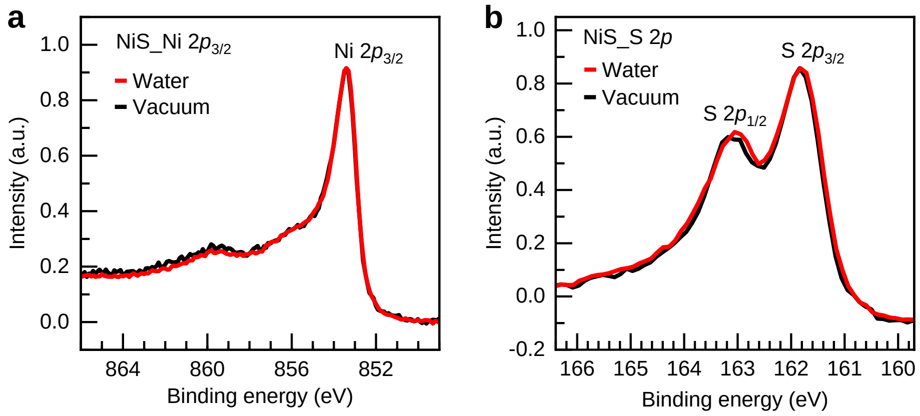

Supplementary Fig. 17 | NAP-XPS measurement under different water vapor pressures. a The Ni 2$p_{3/2}$ XPS spectra of NiS in vacuum and 0.5 mbar water vapor. **b** The S 2*p* XPS spectra of NiS in vacuum and 0.5 mbar water vapor. There is no obvious change in Ni 2$p_{3/2}$ and S 2*p* after water exposure, and the results indicate a very small amount of reaction intermediates (*i.e.*, OH* and H*) can be produced to adsorb on the Ni and S sites, which results from low activity of NiS to dissociate H_2O molecules.

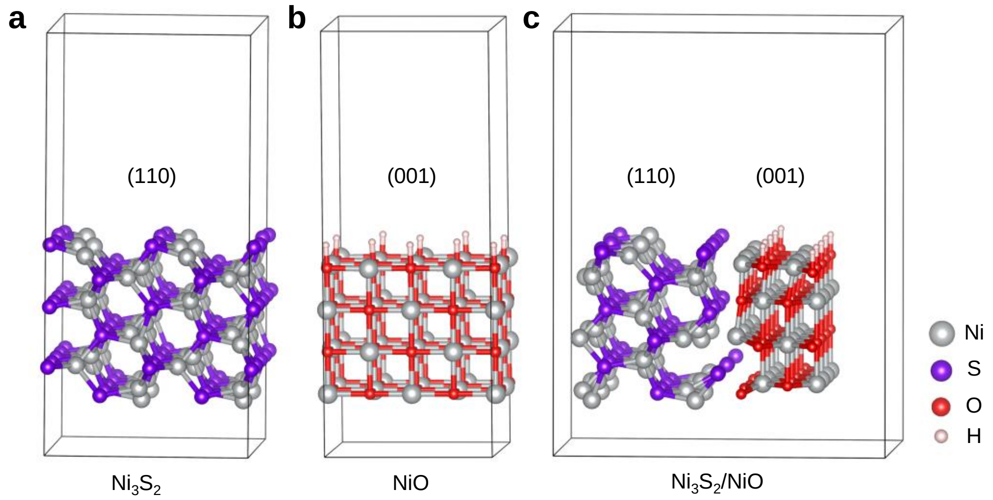

Supplementary Fig. 18 | The constructed theoretical computational model. The optimized structure models for theoretical calculations. **a** Ni_3S_2 (marked as Ni_3S_2); **b** NiO with the O sites covered by H (marked as NiO); **c** Ni heterostructure slab with the O sites covered by H (marked as Ni_3S_2/NiO).

[**Note:** According to experimental and theoretical results, the heterostructure is built through the junction of (1$\bar{1}$0) surface of Ni_3S_2 and (001) surface of NiO obtained from theoretical simulation with lattice mismatch less than 5 %. A vacuum layer with a thickness of 20 Å was built on the surface of the (110) surface of Ni_3S_2 and (001) plane of NiO in heterostructures and oxyhydroxides systems to avoid interactions along the z direction of the supercell. We have employed a heterostructure of Ni_3S_2 and NiO with O sites covered by H as a theoretical model (marked as Ni_3S_2/NiO) for DFT calculations (Supplementary Fig. 18). The Ni_3S_2/NiO model with O sites covered by H can be constructed because the adsorption of H on surface unsaturated oxygen sites for both Ni_3S_2/NiO and NiO is spontaneous and too strong, as revealed by the calculated values of ΔG_{H^*} in Supplementary Fig. 20.]

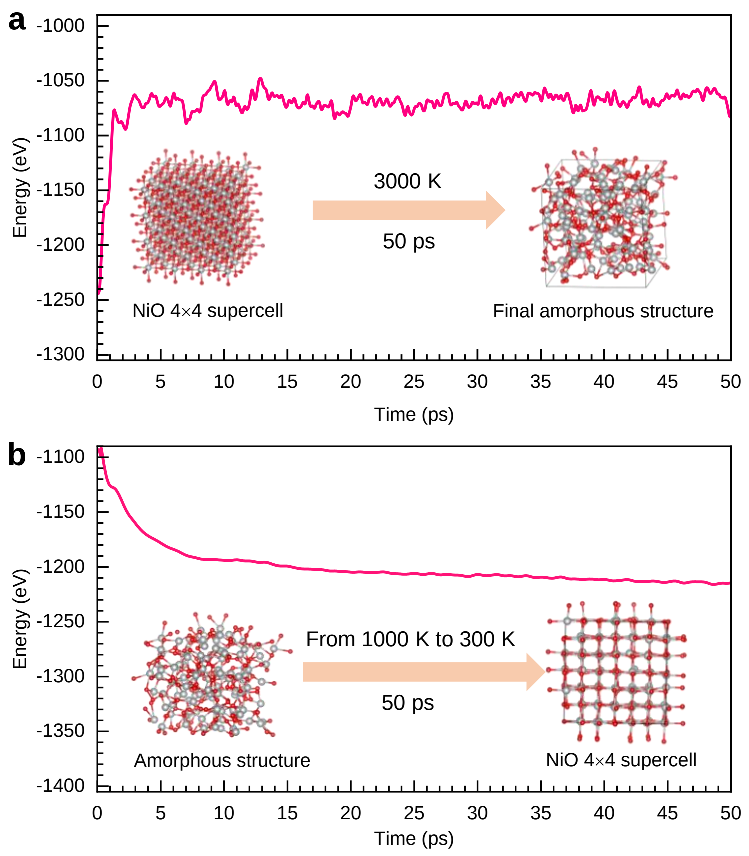

Supplementary Fig. 19 | Molecular dynamics simulations for the rationality of the NiO model. a Ab initio molecular dynamics (AIMD) Nose-Hoover under 3000 K for 50 ps to obtain an amorphous structure of NiO. **b** AIMD Nose-Hoover from 1000 K to 300 K to simulation annealing with NVT ensemble for 50 ps. The result indicates the stable state of NiO is crystalline.

[**Note:** According to the characteristic pattern of XRD, the Ni_3S_2 is determined to be crystalline. For NiO, the combination of XRD, XPS, XAS and Raman results makes it hard to determine whether the NiO is crystalline or amorphous. We can roughly determine the stability of NiO via a thermodynamics analysis. We firstly performed ab initio molecular dynamics (AIMD) to calculate the most likely stable structure of NiO. AIMD calculation of supercell of NiO ($4 \times 4 \times 4$) under the temperature of 3000 K (melting points 2260.15 K) governed by Nose-Hoover thermostat for 50 ps was performed to obtain an amorphous structure (Supplementary Fig. 19a)[13,14]. Afterward, the amorphous structure was optimized under system temperature from 1000 K to 3000 K for 50 ps to simulate the annealing process. Once the temperature was decreased from 1000 K to 300

K (room temperature), it can be found that the amorphous structure would finally convert into a face-centered cubic (FCC) structure (Supplementary Fig. 19b)[15,16]. Therefore, the employed crystalline NiO model would be reasonable. This result consists with the analysis of SAC-TEM. Note that the final NiO model via AIMD and DFT simulation underwent a geometrical optimization process in terms of lattice parameters and ions position.]

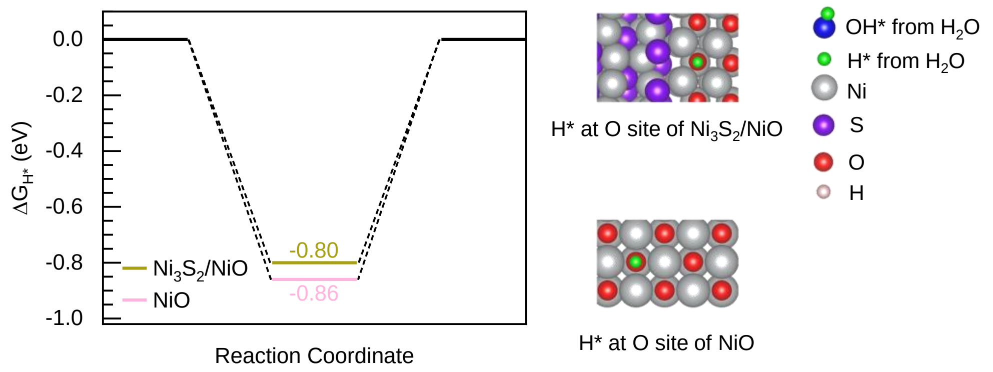

Supplementary Fig. 20 | Theoretical computational results. The calculated Gibbs free energy diagrams of H* adsorption at O sites of Ni_3S_2/NiO and NiO. The results indicate the adsorption of H* on surface unsaturated oxygen sites for both Ni_3S_2/NiO and NiO is spontaneous and too strong.

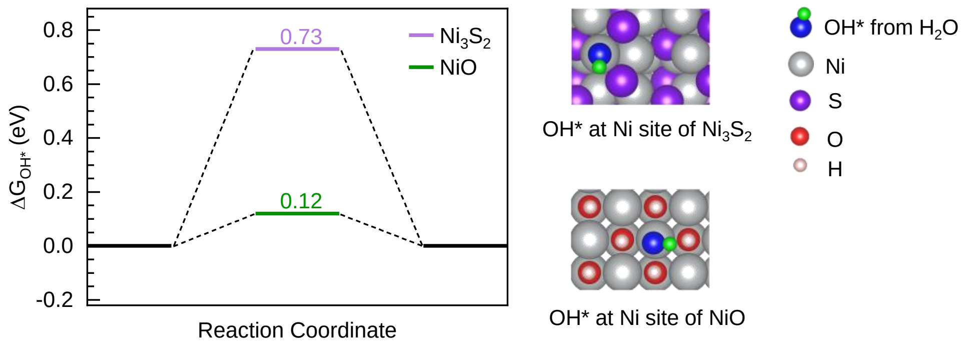

Supplementary Fig. 21 | Theoretical computational results. Gibbs free energy diagrams of OH* adsorption at Ni sites of NiO and Ni_3S_2. The results indicate Ni sites of NiO exhibit stronger adsorption for OH*.

Supplementary Table 8 | The pH values of 1 M KOH electrolyte prepared 5 times, with calculated average and error bar.

Times	1	2	3	4	5	Average	Error bar
pH	13.98	14.01	14.03	14.04	13.96	14.00	±0.03

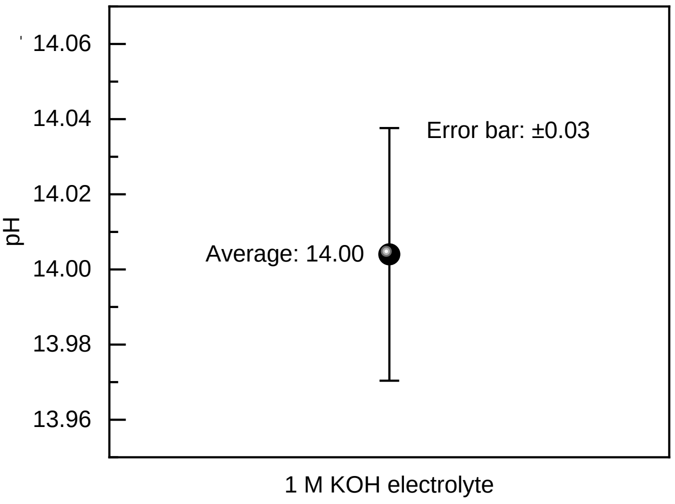

Supplementary Fig. 22 | The pH values of as-prepared 1 M KOH electrolyte. Its data are sourced from Supplementary Table 8.

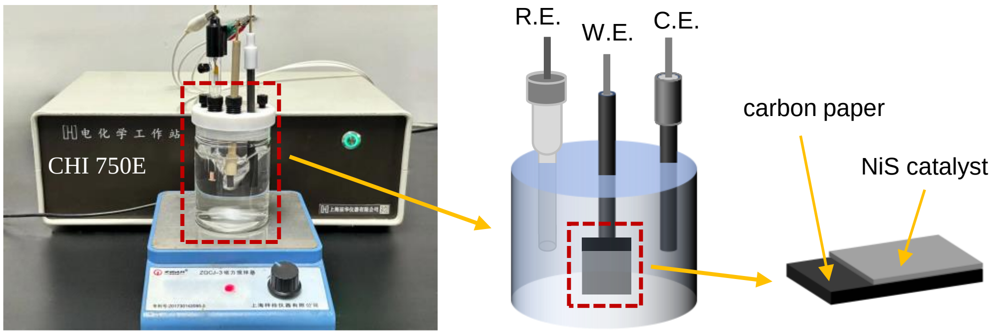

Supplementary Fig. 23 | The experimental set up for electrochemical measurements. It consists of a CHI 750E electrochemical workstation, a three-electrode configuration and a magnetic stirring system. The three-electrode configuration contains working electrode (NiS catalyst), reference electrode (Hg/HgO) and counter electrode (carbon rod). They are abbreviated as W.E., R.E. and C.E., respectively.

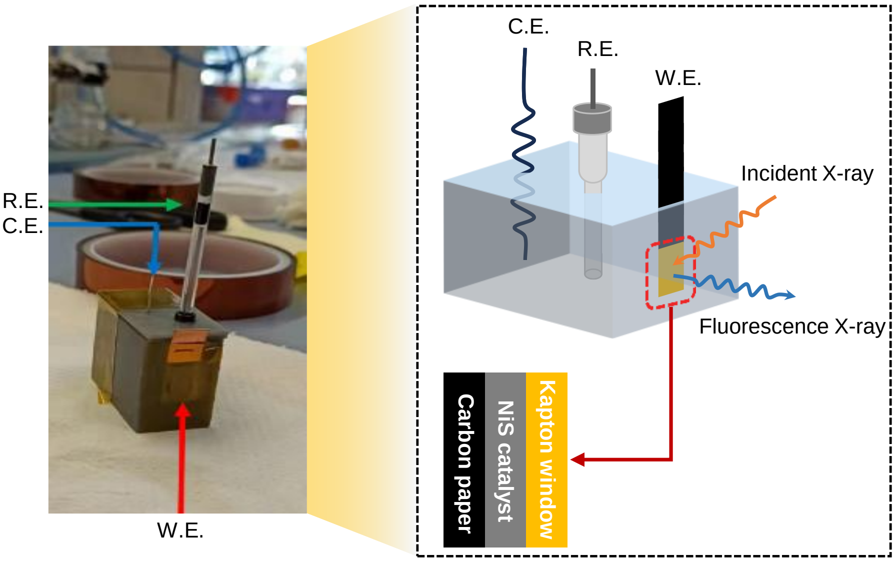

Supplementary Fig. 24 | Electrochemical cell for operando XAS measurements. The experimental set-up for operando XAS measurement. W.E., R.E. and C.E. stand for working electrode (NiS catalyst), reference electrode (Ag/AgCl) and counter electrode (platinum wire), respectively.

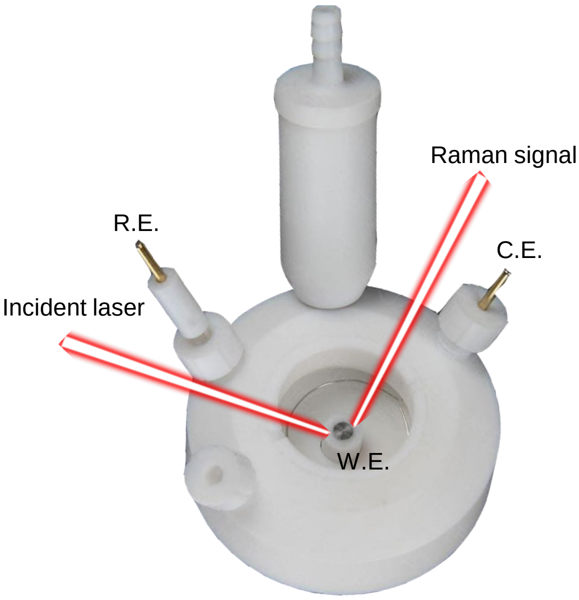

Supplementary Fig. 25 | Electrochemical cell for operando Raman measurements. The experimental set-up for operando Raman measurement. W.E., R.E. and C.E. stand for working electrode (NiS catalyst), reference electrode (Ag/AgCl) and counter electrode (platinum wire), respectively.

Supplementary Notes

Supplementary Note 1

EXAFS simulations procedure

The software utilized for EXAFS analysis is ARTEMIS from the Demeter package. The crystallographic information files (CIFs) for NiS and Ni_3S_2 were employed in the FEFF 6 calculation to derive the scattering paths. The software employs the path expansion approximation, treating the EXAFS spectrum as the sum of contributions from scattering geometries involving two or more atoms in a cluster surrounding the absorbing atom[17]. Before the EXAFS analysis, the data underwent normalization through background subtraction using the ATHENA software within the Demeter package. the EXAFS data were analyzed in the *k*-range of 3–11 $Å^{-1}$, and the Fourier Transform was executed using a Hanning window. The data were fitted in the *R*-range of 1–2.6 Å using a multiple *k*-weight fitting. The fits employed the most intense single scattering paths, Ni-S and Ni-Ni, associated with NiS and Ni_3S_2 phases, in accordance with the results obtained from XRD, Raman, and XANES investigations. When there was evidence of the presence of Ni-O bonds, the single scattering path of NiO was added. We weighted the Ni-S and Ni-O contribution by introducing a parameter x. The amplitude of Ni-S and Ni-O shell was x and 1−x, respectively. The x value was fixed based on LCF fitting results. The amplitude reduction factor S_0^2 was determined from a reference Ni foil, and its value of 0.8 was fixed for all scattering paths. This procedure is widely accepted in the analysis of EXAFS data, particularly when experiments are conducted under the same conditions.

Regarding the model for fitting the data, we would like to clarify as following: 1) only the most intense single scattering paths were utilized, 2) the coordination number of the Ni-Ni shell was fixed based on crystallographic data of NiS or Ni_3S_2, while the coordination number of the first shell was treated as a free parameter. This approach was necessary because, when leaving the coordination number of the second shell as a free parameter in the fit, a high correlation was observed with the Ni-S shell. The contributions of Ni-S and Ni-Ni are included in a single shell and cannot be distinguished within a limited *k*-range. Conversely, R and σ of the first and second shells were treated as

independent parameters.

Supplementary Note 2

Synthesis of Ni_3S_2 reference

The Ni_3S_2 reference was synthesized by heating stoichiometrically mixed elements of nickel and sulfur at 800 °C for 6 hours in a silica tube sealed under vacuum. The XRD patterns shown in Supplementary Fig. 26a confirm that as-synthesized Ni_3S_2 has high crystalline quality. The shapes and positions of Ni 2*p* and S 2*p* XPS spectra shown in Supplementary Figs. 26b and 26c are also the same as previous report of Ni_3S_2[18]. The Ni K-edge spectra for our Ni_3S_2 reference also agrees well with the spectra from the Farrel-Lytle database (Supplementary Fig. 26d), which allows us to confidently use the Ni_3S_2 reference for our analysis.

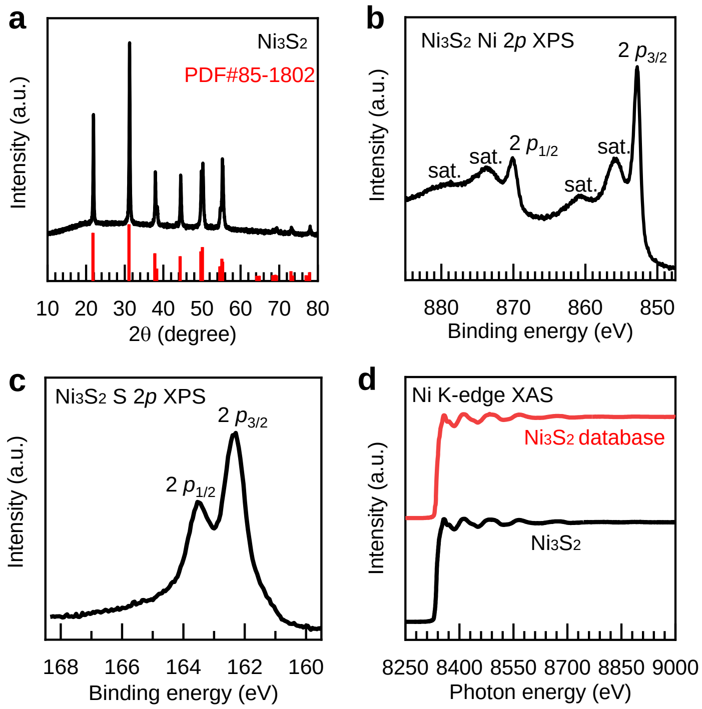

Supplementary Fig. 26 | Characterizations of as-synthesized Ni_3S_2 reference. a XRD patterns of as-synthesized Ni_3S_2 crystal powder and the standard card PDF#85-1802. **b** Ni 2*p* XPS and **c** S 2*p* XPS spectra for as-synthesized Ni_3S_2. **d** Ni K-edge XAS spectra of as-synthesized Ni_3S_2 and Ni_3S_2 spectra from the Farrel-Lytle database (http://ixs.iit.edu/database/data/Farrel_Lytle_data/).

Supplementary Note 3

NiO and Ni_3S_2 reference

The reference sample for NiO was a commercial powder. In order to get the Ni K-edge XAS reference of NiO, a proper amount of NiO sample was weighted, mixed with cellulose and then pressed into a 13 mm pellet for XAS measurement in transmission mode. For Ni_3S_2 reference, it was a crystal powder synthesized by ourselves, and the methods were shown in Supplementary Note 2. In order to get the Ni K-edge XAS reference of Ni_3S_2, proper amount of Ni_3S_2 powder was weighted, mixed with cellulose and then pressed into a 13 mm pellet for XAS measurement in transmission mode. The spectra was aligned with our data by comparison with a Ni foil.

Supplementary Note 4

Principal component analysis

Principal component analysis (PCA) was performed using a set of 6 Ni K-edge spectra measured during the positive sweep potential. Despite PCA is a mathematical tool that cannot explain which phases are present in a certain sample, it can give an estimation of the number of phases present.

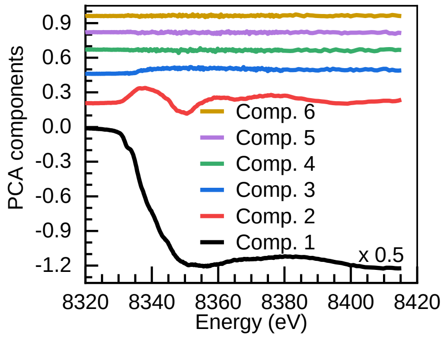

Supplementary Fig. 27 | PCA analysis. Principal component analysis (PCA) of the XAS spectra measured during the positive sweep potential at six different values. The first component (mean) was multiplied by 0.5 for a better comparison.

In our case, as shown in Supplementary Fig. 27, we can see that most of the spectrum is reproduced by comp. 1 (mean spectrum). Additionally, comp. 2 is necessary to explain

the different spectroscopic features observed. Comp. 3 is not giving a relevant improvement of the fit and the cumulative variance is 0.999 after considering only the first two components. The other components are mainly associated with noise. On these bases, we think that the use of two references is reasonable to explain the spectral features in our system.

Supplementary Note 5

Linear combination fitting

Based on PCA, the use of two references is reasonable to explain the spectral features in our system. We have followed reason to believe the two references should be Ni_3S_2 and NiO for linear combination fitting (LCF).

Ni K-edge XAS during a cycle of CV gives adequate reasons for using Ni_3S_2 and NiO as reference. As shown in region II in Supplementary Fig. 28a (Fig. 2c in main text), the similarity of the measured spectra with Ni_3S_2 makes straightforward its use as a reference for the linear combination fitting. When it goes into region III in Supplementary Fig. 28a, NiO was a reasonable reference because the increase of the whiteline of 8350 eV is a typical feature of NiO and the slight shift of the edge towards higher energy indicates the presence of a more electronegative ion. Meanwhile, the XRD of NiS after HER measurement corresponds to Ni_3S_2 (Supplementary Fig. 28b), which indicates it also contains Ni_3S_2 in region III. Moreover, the first shell suffers an appreciable contraction for specific potentials due to the Ni-O bond formation (Supplementary Figs. 12, 13 and Supplementary Table 4). We also detect the formation of NiO by other characterizations (*e.g.*, Raman spectroscopy, XPS and TEM, mentioned in the main text). As shown in Supplementary Fig. 28b, because no XRD pattern was observed for NiO, we think the NiO should be in the form of a very thin layers whether crystalline or amorphous. However, the XANES features of amorphous NiO are very similar to those of crystalline NiO. As reported by R. Li et al.[19], the XANES for amorphous NiO and crystalline NiO are quite similar. For all these reasons, we think that a relevant description of the system may be obtained using Ni_3S_2 and NiO references.

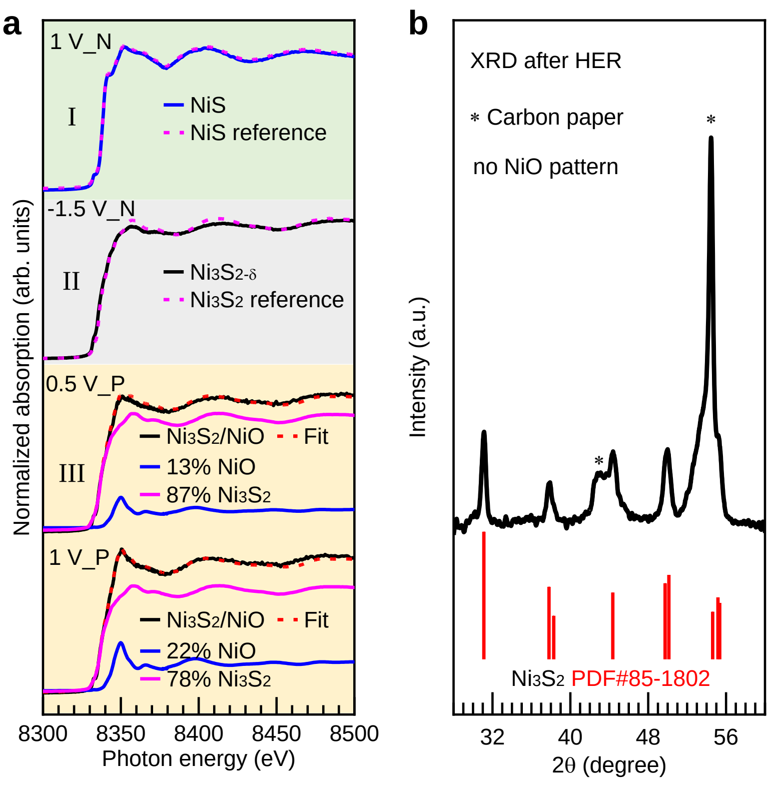

Supplementary Fig. 28 | LCFs analysis of XAS and XRD pattern. a The Ni K-edge XAS spectra at specific applied voltages of +1.00 V (1 V_N means +1.00 V in negative sweep of CV), −1.50 V (−1.50 V_N means −1.50 V in negative sweep of CV), and +0.50 V, +1.00 V (+0.50 V_P and +1.00 V_P mean +0.50 V and +1.00 V in positive sweep of CV) in region I, II and III respectively, and their linear combination of Ni_3S_2 and NiO. **b** XRD pattern for NiS after HER measurement. The pattern was assigned to Ni_3S_2.

Supplementary Note 6

The accuracy of calculations

The crystal structures and electronic structures of NiO and Ni_3S_2 have been previously reported by experiment and theory[20-23]. Therefore, the crystal structure, lattice parameters, atomic bond distances and angles of NiO and Ni_3S_2 are optimized according to the literature and compared to the results of previous works[20], as listed in Supplementary Table 9. The good agreement between the optimized lattice parameters and the experimental results suggests our computational methodology is reliable to describe the crystal structure of NiO and Ni_3S_2.

Supplementary Table 9 | The crystal parameters, internal atomic distances, and angles of NiO and Ni_3S_2.

		Computational results	Experimental results[20,23,24]
NiO (P63/mmc)	(a, b, c)	(4.21, 4.21, 4.21)	(4.18, 4.18, 4.18)
	(α, β, γ)	(90.0, 90.0, 90.0)	(90.0, 90.0, 90.0)
	Ni-O	2.10	2.09
	Ni-Ni	2.97	2.96
	< O-Ni-O	(90.0, 180.0)	(90.0, 180.0)
Ni_3S_2 (R32H)	(a, b, c)	(5.69, 5.69, 7.08)	(5.67, 5.67, 7.05)
	(α, β, γ)	(90.0, 90.0, 120.0)	(90.0, 90.0, 120.0)
	Ni-S	(2.24, 2.27)	(2.24, 2.26)
	Ni-Ni	2.51	2.50
	< S-Ni-S	(100.5, 127.7)	(100.8, 127.3)
	< Ni-Ni-Ni	(60.0, 98.9, 108.2, 38.4)	(60.0, 99.0, 108.1, 38.3)

For the electronic structure, previous experimental and theoretical studies reported that NiO has semiconductor states[25] and Ni_3S_2 has metallic states[21,22]. Our DFT calculated electronic structures for NiO (Supplementary Fig. 29a) and Ni_3S_2 (Supplementary Fig. 29b) also indicate the semiconductor state and metallic state, respectively. Furthermore, the calculated density of state (DOS) also indicates that there are substantial Ni 3*d* states contributing to the DOS, also in good agreement with the literatures[21,22,25]. Therefore, our calculation methods can reproduce well the structural and electronic properties of NiO and Ni_3S_2.

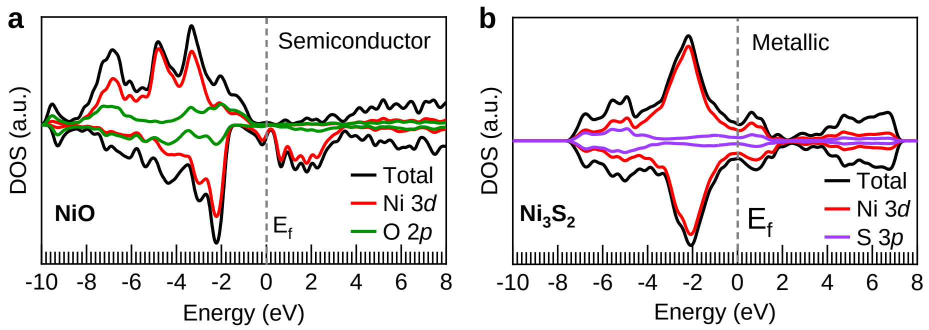

Supplementary Fig. 29 | The DOS calculated by DFT. Calculated total and partial density of states of samples: **a** NiO and **b** Ni_3S_2.

We also calculated the HER steps on NiO and Ni_3S_2 and compared the data with other published work[26,27], which was performed by using PBE functional. Our results are well consistent with the reference data[26,27]. The usage of this method (GGA-PBE) in our work shows very good performance in understanding the reaction mechanism and activity trends observed in experiments. Therefore, the accuracy of DFT calculations used in our work is reliable.

Supplementary References

1. McCrory, C. C. L., Jung, S., Peters, J. C. & Jaramillo, T. F. Benchmarking heterogeneous electrocatalysts for the oxygen evolution reaction. *J. Am. Chem. Soc.* **135**, 16977-16987 (2013).
2. Xu, K. *et al.* Regulating water-reduction kinetics in cobalt phosphide for enhancing HER catalytic activity in alkaline solution. *Adv. Mater.* **29**, 1606980 (2017).
3. Jiang, J., Gao, M., Sheng, W. & Yan, Y. Hollow chevrel-phase $NiMo_3S_4$ for hydrogen evolution in alkaline electrolytes. *Angew. Chem. Int. Ed.* **55**, 15240-15245 (2016).
4. Zheng, Y.-R. *et al.* Doping-induced structural phase transition in cobalt diselenide enables enhanced hydrogen evolution catalysis. *Nat. Commun.* **9**, 2533 (2018).
5. Feng, J.-X., Wu, J.-Q., Tong, Y.-X. & Li, G.-R. Efficient hydrogen evolution on Cu nanodots-decorated Ni_3S_2 nanotubes by optimizing atomic hydrogen adsorption and desorption. *J. Am. Chem. Soc.* **140**, 610-617 (2018).
6. Rahman, G., Chae, S. Y. & Joo, O.-s. Efficient hydrogen evolution performance of phase-pure NiS electrocatalysts grown on fluorine-doped tin oxide-coated glass by facile chemical bath deposition. *Int. J. Hydrogen Energy* **43**, 13022-13031 (2018).
7. Tian, T., Huang, L., Ai, L. & Jiang, J. Surface anion-rich NiS_2 hollow microspheres derived from metal–organic frameworks as a robust electrocatalyst for the hydrogen evolution reaction. *J. Mater. Chem. A* **5**, 20985-20992 (2017).
8. Yin, J. *et al.* Atomic arrangement in metal-doped NiS_2 boosts the hydrogen evolution reaction in alkaline media. *Angew. Chem. Int. Ed.* **58**, 18676-18682 (2019).
9. He, W. *et al.* Fluorine-anion-modulated electron structure of nickel sulfide nanosheet arrays for alkaline hydrogen evolution. *ACS Energy Lett.* **4**, 2905-2912 (2019).
10. Faber, M. S. *et al.* High-performance electrocatalysis using metallic cobalt pyrite (CoS_2) micro- and nanostructures. *J. Am. Chem. Soc.* **136**, 10053-10061 (2014).
11. Wang, W. *et al.* A self-supported $NiMoS_4$ nanoarray as an efficient 3D cathode for the alkaline hydrogen evolution reaction. *J. Mater. Chem. A* **5**, 16585-16589 (2017).
12. Yu, Z.-Y. *et al.* A one-dimensional porous carbon-supported Ni/Mo_2C dual catalyst for efficient water splitting. *Chem. Sci.* **8**, 968-973 (2017).

13. Zhao, X., Ceresoli, D. & Vanderbilt, D. Structural, electronic, and dielectric properties of amorphous ZrO_2 from ab initio molecular dynamics. *Phys. Rev. B* **71**, 085107 (2005).

14. Lee, T. H. & Elliott, S. R. Ab initio computer simulation of the early stages of crystallization: application to $Ge_2Sb_2Te_5$ phase-change materials. *Phys. Rev. Lett.* **107**, 145702 (2011).

15. Qiao, M., Liu, J., Wang, Y., Li, Y. & Chen, Z. $PdSeO_3$ monolayer: promising inorganic 2D photocatalyst for direct overall water splitting without using sacrificial reagents and cocatalysts. *J. Am. Chem. Soc.* **140**, 12256-12262 (2018).

16. Branicio, P. S. *et al.* Atomistic insights into the nanosecond long amorphization and crystallization cycle of nanoscale $Ge_2Sb_2Te_5$: An ab initio molecular dynamics study. *Physical Review Materials* **2**, 043401 (2018).

17. Ravel, B. Path aggregation techniques for EXAFS visualization and analysis. *Journal of Physics: Conference Series* **430**, 012006 (2013).

18. Buckley, A. N. & Woods, R. Electrochemical and XPS studies of the surface oxidation of synthetic heazlewoodite (Ni_3S_2). *J. Appl. Electrochem.* **21**, 575-582 (1991).

19. Li, R. *et al.* Short-range order in amorphous nickel oxide nanosheets enables selective and efficient electrochemical hydrogen peroxide production. *Cell Reports Physical Science* **3**, 100788 (2022).

20. Vershinin, A. D., Selivanov, E. N., Gulyaeva, R. I. & Sel'menskikh, N. I. Thermal expansion of Ni_3S_2 in Ni_3S_2-Ni alloys. *Inorg. Mater.* **41**, 882-887 (2005).

21. Wang, J.-H., Cheng, Z., Bredas, J.-L. & Liu, M. Electronic and vibrational properties of nickel sulfides from first principles. *J. Chem. Phys.* **127**, 214705/214701-214705/214708 (2007).

22. Lu, Z. W., Klein, B. M. & Singh, D. J. Electronic structure of heazlewoodite Ni_3S_2. *Phys. Rev. B: Condens. Matter* **54**, 13542-13545 (1996).

23. Smith, N. The structure of thin films of metallic oxides and hydrates. *J. Am. Chem. Soc.* **58**, 173-179 (1936).

24. Parise, J. Structure of hazelwoodite (Ni_3S_2). *Acta Crystallographica Section B* **36**,

1179-1180 (1980).

25. Fu, G. *et al.* Tuning the electronic structure of NiO via Li doping for the fast oxygen evolution reaction. *Chem. Mater.* **31**, 419-428 (2019).

26. Feng, L.-L. *et al.* High-index faceted Ni_3S_2 nanosheet arrays as highly active and ultrastable electrocatalysts for water splitting. *J. Am. Chem. Soc.* **137**, 14023-14026 (2015).

27. Zhao, L. *et al.* Steering elementary steps towards efficient alkaline hydrogen evolution via size-dependent Ni/NiO nanoscale heterosurfaces. *National Science Review* **7**, 27-36 (2020).
