# Supplementary material for: Dynamic restructuring of nickel sulfides for electrocatalytic hydrogen evolution reaction
Source: Nat Commun. 2024 Jun 24;15:5336. doi: 10.1038/s41467-024-49015-4 (PMC11196257; doi:10.1038/s41467-024-49015-4)
Supplement: Supplementary file 3 — Description of Additional Supplementary Files [file 41467_2024_49015_MOESM3_ESM.pdf]

Description of Additional Supplementary Files

Supplementary Data 1 : The atomic coordinates of the optimized computational models used for electronic structure calculations and molecular dynamics simulations reported in the manuscript.
